# Proximal femur versus acetabular extra-articular resection of the hip joint for primary malignant bone tumors: a retrospective comparative review of 33 cases

**DOI:** 10.1186/s12957-022-02642-x

**Published:** 2022-05-28

**Authors:** Victor Housset, Philippe Anract, Antoine Babinet, Guillaume Auberger, David Biau

**Affiliations:** 1 Service de Chirurgie Orthopédique et Traumatologique, Hôpital Cochin, 75013 Paris, France; 2 Service de Chirurgie Orthopédique et Traumatologique, Hôpital Henri Mondor, 94000 Créteil, France

## Abstract

**Introduction:**

Extra-articular resection (EAR) of the hip joint is prone to significant complications and morbidity. Thus, this study evaluates the cumulative incidences and main reasons of reoperation following EAR of primary malignant bone tumors (PMBT) of the hip to determine whether the outcomes are different between EAR of the pelvis and that of the proximal femur.

**Patients and methods:**

Thirty-three patients presented with a PMBT of the proximal femur or pelvis were included in this study. Among all PMBTs, 58% originated from the pelvis and 42% were from the proximal femur. Twenty patients had chondrosarcomas (61%), 10 had osteosarcomas (30%), and 3 had sarcomas of another histological subtype (9%).

**Results:**

The mean follow-up was of 76 months (range: 24–220 months). The cumulative probabilities of revision for any reason were 52% (95% confidence interval [CI] 30–70%) 5 years after surgery. The 5-year cumulative probabilities of revision were 13% (95% CI 4–27%), 24% (95% CI 10–42%), and 34% (95% CI 14–56%) for mechanical, infectious, and tumoral reasons, respectively. The 5-year cumulative probabilities of revision for any reason were 78% (95% CI 37–94%) and 14% (95% CI 2–38%) for the pelvis and proximal femur, respectively (*p* = 0.004). Posterior column preservation was significantly associated with more mechanical complications even after adjusting for the resection site (*p* = 0.043).

**Conclusion:**

Half of patients undergoing EAR of the hip joint for PMBT of the proximal femur or acetabulum will require another operation. EAR of the pelvis is associated with significantly worse outcome than EAR of the proximal femur.

## Introduction

Bone sarcomas are rare cancers, accounting for less than 0.1% of all cancers [1]. The proximal distal femur, proximal tibia, proximal humerus, and pelvis are the most common anatomical sites for bone sarcomas. Osteosarcoma, chondrosarcoma, and Ewing sarcoma are the most common histologies. The surgical control of a bone sarcoma requires resection of the affected bone with only an approximately 1–2-cm margin at the bony cuts [2] and may require an extensive soft-tissue resection depending on the size and location of the tumor. Despite the development of imaging techniques and increase in the effectiveness of chemotherapy, none of the many reconstructive options described has been established nowadays as a *gold standard* procedure following resection of periacetabular primary malignant bone tumors (PMBTs) due to the challenging aspect of these procedures and the high rate of postoperative complications. However, when a bone sarcoma invades a joint, an extra-articular resection (EAR) (viz. en bloc resection) of the affected bone, joint, and part of the adjacent bone should be performed to obtain adequate margins [3].

EAR of the joint decreases the risk of local recurrence when joint contamination is suspected [4]. Before surgery, the necessary means must be employed to rule out joint contamination. A significant joint effusion on clinical examination should be considered a potential joint contamination. Imaging studies, including MRI, radiography, and CT, are paramount to suspect an invasion of the joint by the tumor, and eventually, biopsy should be performed through the joint.

EARs of the joint are technically challenging and prone to significant complications and morbidity [5]. The most commonly performed EAR of the joint is that of the knee joint with a bone sarcoma affecting either the proximal tibia or the distal femur [6–8]. When performed in an expert centre, EAR of the joint is associated with adequate margins and little local recurrence. EAR and wide resection of the hip joint have been reported in few series [4, 5, 9], without a significant difference in outcomes between resections of PMBTs located in the pelvis and proximal femur. Deep infection, mechanical failure, and local recurrences seem to be the main reasons for revision and reoperation following this surgically demanding procedure [4, 5, 9]. Given the limited information available on this difficult technique, we decided to report our experience to provide information on the technical aspects and expected results of such a procedure.

Therefore, this study evaluates the cumulative incidences of reoperation following EAR of the hip and determines the main reasons for reoperation to compare the different outcomes between EAR of the pelvis and EAR of the proximal femur.

## Patients and methods

### Study design, setting, and participants

This retrospective study involved patients treated between 2000 and 2016 at a tertiary care centre specializing in treating musculoskeletal tumors. Patients who presented with a PMBT of the proximal femur or pelvis and were treated with EAR of the hip joint for articular invasion were included in this study. Those who were <15 years of age, those who presented with a local recurrence after a previous operation and those who presented a non-malignant primitive bone tumor were excluded from the study. All eligible patients, identified using our electronic hospital records, were included. Treatment decisions were discussed at a multidisciplinary team meeting; four senior surgeons with specific expertise in musculoskeletal orthopedic oncology performed all surgeries.

### Surgical technique and postoperative care

A prophylactic antibiotic therapy including metronidazole and cefazolin for 24 h was started at the time of anesthetic induction. The patients were placed on the lateral recumbent position to allow for the necessary surgical exposures, and the surgical approach varied depending on the tumor location. Proximal femoral tumors were approached through a single posterolateral incision (*n* = 7) or both posterolateral and inguinal incisions (*n* = 7). Pelvic tumors were approached through a utilitarian incision (*n* = 7) or both posterolateral and ilio-inguinal incisions (*n* = 12). For both femoral and pelvic tumors, the involved bone and soft tissue were dissected based on oncological principles that a 1–2-cm margin should be retained when possible depending on the extent of the tumor. For proximal femoral tumors, the posterior column, the superior part of the acetabulum, and the iliopubic ramus were freed from soft-tissue attachments, and osteotomies were performed the same way as a periacetabular osteotomy; the acetabulum was then mobilized together with the proximal femur, and the specimen was removed and sent to pathology. In one case, the posterior column was not preserved (7%). For pelvic tumors, the proximal femur was exposed, and a vertical osteotomy in the plane of the greater trochanter was performed, and a horizontal, or short oblique, osteotomy was performed at the upper level of the lesser trochanter; the femoral neck and joint capsule were mobilized together with the pelvic resection, and the specimen was removed and sent to pathology. The posterior column was preserved in two cases (10.5%).

**Fig. 1:**
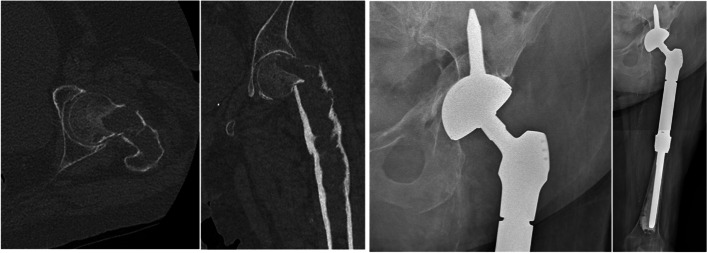
On the left, proximal femur pathologic fracture on a chondrosarcoma. On the right, extraarticular resection and reconstruction with an endoprosthetic reconstruction and ice-cream cone prosthesis performed for the same patient. The posterior column was preserved

**Fig. 2:**
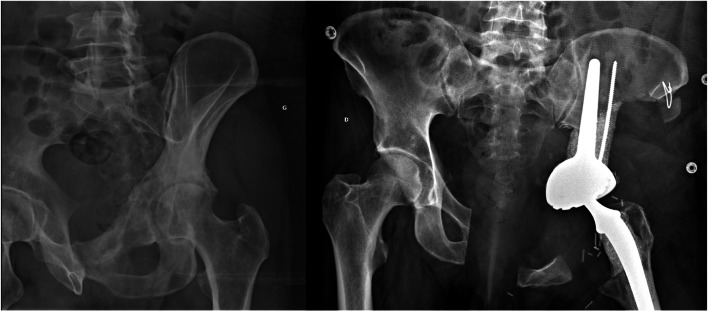
On the left, zone 2 and 3 pelvis chondrosarcoma. On the right, extraarticular resection and reconstruction with an ice-cream cone prosthesis and standard cemented femoral stem performed for the same patient

For every patient, reconstruction was performed simultaneous to the resection. Proximal femoral resections were reconstructed using endoprostheses in five cases (Fig. 1), using allograft–prosthesis composites in six cases and using a standard stem in three cases. On the pelvic side, an ice-cream cone reconstruction was performed in 10 cases (Fig. 2), an acetabular cemented reconstruction with an allograft and acetabular ring was performed in three cases, and an uncemented reconstruction in one case. Pelvic resections were reconstructed using an ice-cream cone in eight cases [10], a saddle prosthesis in four cases, an acetabular cemented reconstruction with an allograft in one case, and no reconstruction (medialisation) in six cases. A standard stem was used in all cases where a saddle prosthesis was not used (15 cases).

Postoperative care included prophylactic anticoagulation for 6 weeks, standard wound care, and removal of the suction drains between postoperative days 2 and 5. For 28 patients (85%), bed rest was maintained for 7–15 days with a transtibial suspension, while wound care was performed in bed. All but one patient had a fibreglass hip spica applied until the sixth postoperative week. Weight bearing was resumed depending on the reconstruction performed. For five patients (15%), bed rest was continued until the patient was comfortable enough to mobilize, and weight bearing was resumed immediately. The standardized postoperative follow-up includes clinical and imaging evaluation (pelvic and hip radiography and pelvic CT or MRI and chest CT) 6 weeks after surgery, then every 4 months during the first 2 years, every 6 months until the fifth year and every year after that.

### Variables, data sources, and measurements

Demographic information, clinical findings, and treatment details were retrieved from the patients’ medical records. All operating notes and pathology reports were reviewed. Histology was defined by one of two pathologists specializing in the bone and soft-tissue tumors from the resection specimen using the residual tumor (R) classification: R0, no residual tumor; R1, microscopic residual tumor; and R2, macroscopic residual tumor. Radiographic contamination was suspected based on the following criteria: an intra-articular fracture of the affected bone or direct visualization of the tumor in the joint. Histological articular contamination was then confirmed, or not, from the resection specimen. The patients’ status was that reported at the time of last review. The following criteria were used for the diagnosis of surgical site infection: A definite surgical site infection was considered if there was a sinus tract communicating with the surgical site; if a pathogen was isolated by culture from at least two separate tissue or fluid samples obtained from the affected prosthetic joint/surgical site; or based on clinical, biologic, and histopathologic exams under review by the multidisciplinary infectious (MDI) board.

These criteria are in accordance, although not strict, with previous published recommendations [11]. However, given the large time span of the study, from 2000 to 2016, we did not adhere to a specific publication guideline; moreover, all infections, or suspected infections, were reviewed by our MDI board.

Mechanical failure included aseptic loosening and hip dislocation requiring open reduction. Local tumor recurrence was usually suspected on imaging follow-up studies and confirmed using percutaneous biopsy. A reoperation was defined as any surgical procedure performed under general anesthesia of the previous surgical site and included open hip reduction. No complication or revision surgeries were excluded in the final analysis even if they occurred less than 1 year postoperatively. Investigations were conducted in accordance with the ethical standards of the 1964 Declaration of Helsinki. The ethical approval was not required according to the MR-004 reference methodology; the study was registered in the *Commission nationale de l'informatique et des libertés* database register (n°2215812), and each patient was individually informed and consented before any data collection [12]. Analyses used R software (R Foundation for Statistical Computing, Vienna, Austria). The significance threshold was set at *p* < 0.05.

### Statistical analysis and data collection

Continuous data are presented as median with the first and third quartile values (Q1–Q3); categorical data are presented as counts and proportions. The cumulative incidence rates of reoperation for infection, mechanical failure, and any reason and the cumulative incidence rate of local recurrence were presented as defined by Prentice et al. [13]. The cumulative incidence rates of events were estimated from the date of EAR of the hip to either the event, competing event (death), or last news when neither event nor death was experienced (censored observation). Point estimates with 95% exact confidence intervals (CIs) are reported. Patient survival was estimated according to the Kaplan–Meier method [14] from the date of EAR of the hip to either death or last news for living patients (censored observation). The log-rank test was used to test for differences between categories of a variable in survival times.

## Results

The median follow-up period was 76 months with a minimum follow-up period of 24 months and a maximum follow-up period of 220 months. In total, 33 patients (Table 1) were enrolled in this study, including 14 women and 19 men, with a median age of 52 years (interquartile range (IQR), 44–61 years). Nineteen tumors originated from the pelvis (58%), and 14 tumors were from the proximal femur (42%). For the 19 pelvic tumors, in three cases, the lesions were type I+II (16%); in ten cases, the lesions were type II+III (52%); and in six cases, the lesions were type I+II+III (32%), according to Enneking and Dunham [15]. Among the 33 lesions, 20 were chondrosarcomas (61%), 10 were osteosarcomas (30%), and three were sarcomas of another histological subtype (9%). Thirteen tumors were high grade (39%). The median size in the larger axis of the bone tumors was 83.7 ± 38.2 mm (IQR: 50–100 mm). Two patients (6%) presented with metastatic disease at the time of surgery. Ten patients had preoperative chemotherapy (30%), and two had preoperative radiation (6%). Nine patients presented with a pathological fracture (27%). Articular contamination was suspected on imaging in 28 patients (85%), and among them, 13 (93%) presented with proximal femur bone tumors (93%) and 15 (79%) presented with periacetabular localisation.

**Table 1:** Characteristics of the population

Characteristics	Total (*n*=33)	Femur (*n*=14)	Pelvis (*n*=19)	*p* value
Median age (Q1–Q3)	52 (44 to 61)	52 (42 to 58)	52 (45 to 62)	0.99
ASA Physical Status Score				0.65
I	15 (46)	5 (36)	10 (52)	
II	12 (36)	6 (43)	6 (32)	
III	6 (18)	3 (21)	3 (16)	
Localization				-
Pelvis	19 (58)	-	19 (100)	
Proximal femur	14 (42)	14 (100)	-	
Enneking and Dunham				-
I–II	3 (16)	-	-	
I–II–III	6 (32)	-	-	
II–III	10 (52)	-	-	
Average size in mm (Q1-Q3)	80 (50 to 110)	85 (49 to 128)	79 (52 to 100)	0.37
Histology				0.11
Chondrosarcoma	20 (61)	6 (43)	14 (74)	
Ewing sarcoma	1 (3)	-	1 (5)	
Ostéosarcoma	10 (30)	7 (50)	3 (16)	
Other sarcoma	2 (6)	1 (7)	1 (5)	
Preoperative metastasis				0.17
No	31 (94)	12 (86)	19 (100)	
Yes	2 (6)	2 (14)	0 (0)	
Preoperative radiation				1
No	31 (94)	13 (93)	18 (95)	
Yes	2 (6)	1 (7)	1 (5)	
Preoperative chemotherapy				0.057
No	23 (70)	7 (50)	16 (84)	
Yes	10 (30)	7 (50)	3 (16)	
Articular contamination (imaging)				0.37
No	5 (15)	1 (7)	4 (21)	
Yes	28 (85)	13 (93)	15 (79)	

*ASA* Physical Status Score: American Society of Anaesthesiologists physical status classification

**Fig. 3:**
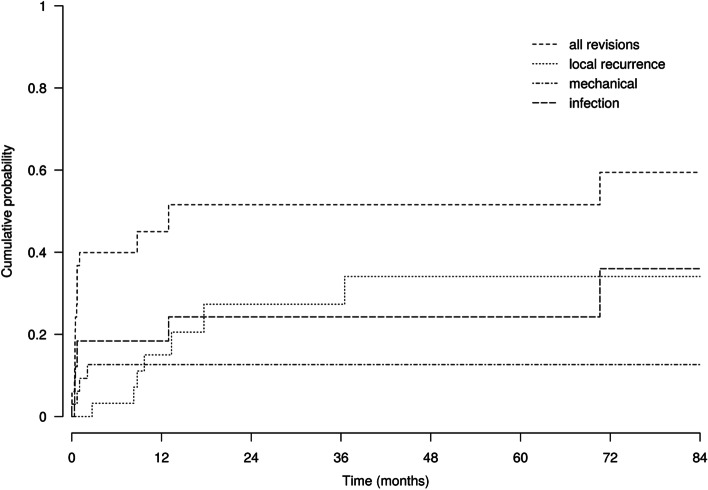
Cumulative incidence of revision for any reason, for mechanical reason, for infection, and local recurrence

The cumulative probabilities of revision for any reason were 45% (95% CI 26–62%), 52% (95% CI 30–70%), and 52% (95% CI 30–70%) one, 2 and 5 years after surgery, respectively. The cumulative probabilities of revision for mechanical reasons were 13% (95% CI 4–27%), 13% (95% CI 4–27%), and 13% (95% CI 4–27%) 1, 2, and 5 years after surgery, respectively. The cumulative probabilities of revision for infection were 18% (95% CI 7–33%), 24% (95% CI 10–42%), and 24% (95% CI 10–42%) 1, 2, and 5 years postoperatively, respectively. The cumulative probabilities of local recurrence were 15% (95% CI 4–31%), 27% (95% CI 10–48%), and 34% (95% CI 14–56%) 1, 2, and 5 years postoperatively, respectively. The cumulative incidences of the main reasons for reoperation after EAR of the hip are represented in Fig. 3.

**Fig. 4:**
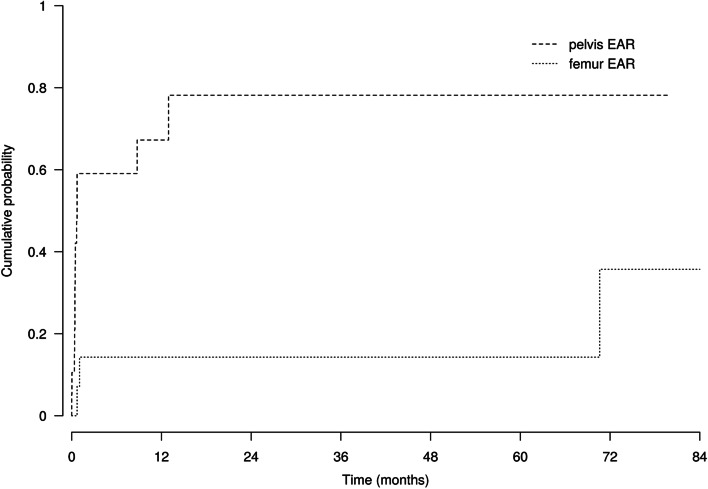
Cumulative incidences of revision for pelvis EAR and proximal femur EAR

Significant differences in outcomes were observed between EAR of the pelvis and EAR of the proximal femur. The 5-year cumulative probabilities of revision for any reason were 78% (95% CI 37–94%) and 14% (95% CI 2–38%) for EAR of the pelvis and EAR of the proximal femur, respectively (*p* = 0.004). The 5-year cumulative probabilities of revision for mechanical reasons were 12% (95% CI 2–32%) and 14% (95% CI 2–38%) for EAR of the pelvis and EAR of the proximal femur, respectively (*p* = 0.8). The 5-year cumulative probabilities of revision for infection were 41% (95% CI 16–64%) and 0% for EAR of the pelvis and EAR of the proximal femur, respectively (*p* = 0.05). The 5-year cumulative probabilities of local recurrence were 54% (95% CI 20–80%) and 29% (95% CI 6–58%) for tumors of the pelvis and proximal femur, respectively (*p* = 0.03). Differences between EAR of the pelvis and EAR of the proximal femur are shown in Fig. 4. The preservation of the posterior column was significantly associated with more mechanical complications, including dislocation and aseptic loosening, even after adjusting for the resection site (pelvis or femur) (hazard ratio [HR] = 0.05; 95% CI = 0.003–0.91; *p* = 0.043); when performing the analysis among patients who had a reconstruction (*n*=23), the effect remained similar but was not significant (hazard ratio [HR] = 0.13; 95% CI = 0.008–2.2; *p* = 0.16).

### Other relevant findings

There was no correlation between the tumor grade, the surgical approaches, and the method of reconstruction with the final outcomes for both techniques. The median surgery length was 300 min (Q1–Q3: 230–360). A mean number of four packed red blood cells were transfused (Q1–Q3: 3–5). Significant intraoperative complications including injuries to the internal iliac vein (*n* = 1), femoral vein (*n* = 1), and ureter (*n* = 1) were observed. All injuries were repaired during the procedure. Accidental intraoperative joint effraction occurred in eight patients: two (11%) during EAR of the pelvis (capsule dissection) and six (43%) during EAR of the proximal femur (bone osteotomies in two cases and capsule dissection in four cases); this difference was statistically significant (*p* = 0.047). Among them, only one (12.5%) has had a revision surgery for hindquarter amputation 3 months after the initial resection. Despite these patients, R0 resection was confirmed in 91% of the patients (30/33) after histological examination of the specimens. At the last follow-up, seven patients passed away, and the 5-year survival probability was 76% (61–95%). The Table 2 summarizes the number and ratio of the encountered complications at the last follow up for the pelvis and proximal femur tumors.

**Table 2:** Total number and ratio for each complication encounter from the procedure to the last follow-up for total population, proximal femur, and pelvis tumors, respectively

	Total (*N* = 33)			Pelvis (*N*= 19)			Femur (*N*= 14)		
	No. complication	No. patient	% (range)	No. complication	No. patient	% (range)	No. complication	No. patient	% (range)
Revision surgeries any causes	29	17	51.5 %	22	13	68.4 %	7	4	28.6%
Mechanical complication	6	5	15.1 %	2	2	10.5 %	4	3	21.4 %
Dislocation reduced under anesthesia	3	3	9 %	0	0	0	3	3	21.4 %
Total dislocation	13	9	27.3 %	3	3	15.8 %	10	6	42.9%
Other mechanical revision	3	2	6 %	2	2	10.5 %	1	1	7.1 %
Total infection	12	9	27.3 %	10	8	42.1 %	2	1	7.1%
Wound surgeries	3	2	6 %	3	2	10.5 %	0	0	0 %
Deep infection	9	7	21.2 %	7	6	31.6 %	2	1	7.1 %
Local recurrence	7	4	12.1 %	7	4	21 %	0	0	0 %
Other surgeries	4	4	3 %	1	1	5.3 %	0	0	0
Neurological complication	2	2	6 %	2	2	10.5 %	0	0	0%
Hematoma	1	1	3 %	1	1	5.3 %	0	0	0
Parietal complication	1	1	3 %	1	1	5.3 %	0	0	0
Oncologic surgery	7	4	15.1 %	7	4	21 %	0	0	0 %
Per-operative complication	8	5	15.1 %	7	4	21 %	1	1	7.1%
Capsular effraction	8	8	24.2 %	2	2	10.5 %	6	6	42.9 %
Non R0 margin	3	3	9%	3	3	15.8%	0	0	0 %

## Discussion

In the treatment of periacetabular bone tumors, a wide variety of treatment possibilities exist depending on the type of lesion. In case of locally aggressive bone tumor, for example in case of chondroblastoma located in the epiphysis of the proximal femur, an intralesional curettage with cement or synthetic bone substitute to fill the bone defect can be used with good results [16]. For some tumors with important local extension, amputation may be indicated with its own morbidity. Sometimes, it may also be possible to proceed to other reconstruction strategy as a plastic lengthening amputation with vascularized bone graft to preserve the knee joint function in selected bone sarcoma patients and for fixing the artificial limb well [17]. When a bone sarcoma invades a joint, EAR should be performed to obtain adequate margins to decrease the risk of local recurrence [4, 6, 7, 18]. EARs of the joint are technically challenging and prone to significant complications and morbidity [4, 6–8, 18]. Although the technique is well known for the knee joint, fewer information exists with regard to its use in the hip joint [4, 5, 9]. For periacetabular bone tumors, an image-guided core biopsy and immunohistochemistry over a fine needle aspiration cytology are recommended to prevent any mis diagnosis that may lead to an unnecessary surgery for example in case of a primary sacral non-Hodgkin’s lymphoma that may be firstly consider as a metastasis for example [19].

Li and colleagues have reported their experience on 18 patients who presented with either pelvic sarcoma or proximal femur sarcoma and suspicion of joint invasion [4]. They reported one (6%) postoperative death. Wide margins were achieved in 72% of cases, and the proportion of local recurrence was 24% after a mean follow-up period of 35 months. In their series, Li and colleagues did not preserve the posterior column when the tumor originated from the proximal femur. However, Rudiger and colleagues have reported two cases of EAR of the hip joint with preservation of the posterior column [20]. Interestingly, we showed that preserving the posterior column was associated with significantly more mechanical failures. This could be because surgeons tended to use the remaining, probably fragile, posterior column as a means of fixation when it is in fact counterproductive.

A recent nationwide survey in 17 Japanese institutions on 80 prosthetic reconstructions using constrained-type hip tumor prostheses following wide resection of periacetabular tumors with a mean follow-up period of 65 months found a local recurrence rate of 26%, a deep infection rate of 39%, and the occurrence of prosthesis-related complications requiring surgery in 9% of the case [9]. The rates of postoperative complications were still high, but comparable to those found in previous studies. Meanwhile, Fujiwara and colleagues have conducted a retrospective study involving a comparable number of patients (*n* = 34) and found a higher occurrence rate of complications in patients who underwent EAR of the hip joint for bone sarcomas involving the pelvis than that in patients who underwent EAR of proximal femur tumors with a shorter mean follow-up period of 38 months. No statistical differences were observed between pelvic EAR and proximal femur EAR (*p* = 0.116). Therefore, in this study, we described the outcomes of a large retrospective series of EAR of the hip in a single-center specializing in this type of procedures and looked for differences between pelvic EAR and proximal femur EAR with a longer follow-up.

The overall 5-year cumulative probability of revision for any reason was 52% (95% CI 30–70%) with a significant difference between pelvic EAR and proximal femur EAR. The 5-year cumulative probabilities of revision for any reason were 78% following pelvic EAR and 14% following proximal femur EAR. The reoperation rate is therefore high. Hoffmann et al. in a retrospective series of 241 Ewing sarcomas of the pelvis have reported a 20% failure rate [21]. Brown and colleagues in a recent systematic review of the literature published between 1990 and 2017 have analysed 1700 periacetabular oncological lesions with a mean follow-up period of 3.4 years [22]. They found a 50% complication rate. Hillmann and colleagues in a retrospective review of 110 cases have reported a 58% complication rate after pelvic reconstruction [18]. Proximal femoral resection and reconstruction are associated with significantly less complications. In a recent systematic review of 41 studies, Janssen and colleagues have reported a 10% revision rate (ranging from 0 to 69%) for endoprosthetic reconstruction [23]. At their last follow-ups, Ogura et al. and Fugiwara et al. have reported 59% and 69%, respectively, occurrence rates of complications requiring revision. Overall, the revision rate in this study is similar to those found in the literature although the patients in this study may have had more aggressive tumors and undergone more extensive surgeries following the concept of EAR of the whole hip joint.

The local recurrence rate was 54% after pelvic EAR and 29% after proximal femur EAR, which is high after resection of a bone sarcoma. Guo and colleagues in a retrospective study involving a series of 30 patients with osteosarcoma have reported a 26% local recurrence rate [24]. Although their follow-up period was twice as short as ours, the local recurrence rate at the last follow-up in the study by Fujiwara et al. (21%) was less significant for both pelvic (23%) and proximal femoral (13%) locations [5]. Li and colleagues in their retrospective study on a series of 18 patients have reported a local recurrence rate of 18%; however, they did not mention joint invasion [4]. Bus and colleagues in a multicenter retrospective review of 162 primary central chondrosarcomas of the pelvis have reported a 38% local recurrence rate [25]. Similarly, the recurrence rate in this study is higher than that for EAR of the proximal femur. In our own experience of 32 proximal femur resections for bone tumors, the local recurrence rate was 9% [26]. Although the aggressiveness of the tumor could explain these differences, the technical difficulties encountered during the procedure may also be an explanation. Indeed, preoperative joint effraction occurred in eight patients (24%). Interestingly, this complication was significantly more common after proximal femur EAR than after pelvic EAR; the additional procedure for an EAR of the hip is technically more challenging for a femur than for a pelvis. The use of navigation and robotics or the use of patient-specific cutting guides may improve the resection by joint invasion at the bony cuts [27, 28]. As described in their retrospective review on 15 patients who underwent resections for primary tumors of pelvis or sacrum using canulated screws and Gigli saw to facilitate the directional control of the osteotomy, Ji et al. found an ideal resection accuracy with a high likelihood of negative margin resections. This effective method allows a precise control of the osteotomy and allow achievement of planned surgical margins and may be used for this indication [29]. The good functional and oncological outcomes of pasteurized autograft reconstruction after resection of periacetabular malignant bone tumors was demonstrated by Guo et al. with a relatively low incidence of complications. In their series, the mean time of bone union was 12 months after the operation [30].

This study has several limitations. First, a limited number of patients were included. This is due to the low incidence of bone sarcomas of the pelvis or proximal femur with joint contamination. Li and colleagues have analyzed 18 cases only among more than 1500 pelvic and proximal femur tumors [4]. Ogura and colleagues had a larger number of patients but from different centers, which may not ensure the reproducibility of the procedure, and the procedures were not performed in centers specializing in treating primary bone tumors [9]. We included 33 patients over 16 years’ experience at our reference centre, which is a similar number of patients as that in the study by Fujiwara et al. but covering a shorter period. Second, the retrospective design of this study is a limitation to the type of data that may be collected. However, this is a common limitation in such rare conditions. Third, the heterogeneity in patients and the surgical techniques employed for resection or reconstruction are a limitation to providing guidelines after EAR of the hip; however, most procedures included non-constrained prostheses, which may have diminished the rate of mechanical failures of the reconstructions.

## Conclusion

Half of the patients undergoing EAR of the hip joint for PMBTs of the proximal femur or acetabulum will require another operation. Pelvic EAR is associated with significantly worse outcomes than proximal femur EAR. Surgeons should be aware that the procedure is technically challenging and that joint invasion is common. Preserving the posterior column may be attempted, but fixation should not rely on it.

## Data Availability

The datasets used and/or analyzed during the current study are available from the corresponding author on reasonable request.
